# Human platelets express CAR with localization at the sites of intercellular interaction

**DOI:** 10.1186/1743-422X-8-456

**Published:** 2011-09-30

**Authors:** Elena Gupalo, Liudmila Buriachkovskaia, Maha Othman

**Affiliations:** 1 Institute of Experimental Cardiology, Cardiology Research Complex, Moscow, Russia; 2 Department of Biomedical and Molecular sciences, Queen's University, Kingston, Ontario, Canada; 3 Laurentian University- St Lawrence College Collaborative Program, Kingston, Ontario, Canada

## Abstract

Adenovirus has a wide tissue tropism. The virus attaches to the surface of cells via the fiber protein knob binding to the Coxsackie and Adenovirus receptor known as CAR. Virus entry inside cells is facilitated by integrins αVβ3 and αVβ5. Mice platelets are shown to be the predominant Ad binding blood cell type and the virus is documented inside platelets. CAR was identified on human platelets in one study yet contradicted in another. The presence of CAR appears to be the most reasonable initial step for virus entry into platelets and is a key to the understanding of platelet adenovirus interaction. This study aimed to re investigate the presence of CAR on human platelets. Platelets were tested by indirect immune-fluorescence using rabbit H-300 polyclonal anti-CAR antibody and goat anti-rabbit IgG F(ab')_2 _Texas Red antibodies, alongside with CAR positive and negative controls. Platelets were found to express CAR on their surface and in contrast to the previous study only 3.5 ± 1.9% of the tested platelets did express CAR. In addition, CAR was seen within intracellular aggregates localized at the sites of cell-cell contacts indicating that CAR expression might be upregulated in response to platelet stimulation. We confirm the presence of CAR on human platelets, we provide explanation to some of the discrepancies in this regards and we add that this receptor is localized at the sites of intercellular interaction.

## 

Adenovirus primarily attaches to the surface of cells via the fiber protein knob binding to coxsackie and Adenovirus receptor known as CAR [1]. The adhesion molecules integrin αVβ3 [2] and αVβ5 [3] act as co-receptors to facilitate the internalization of virus inside these cells where Ad interacts to these molecules via the RGD-containing Ad penton base protein. In addition to αVβ3 and αVβ5, other integrin such as αVβ1, and α5β1 have also been reported to bind Ad [4].

Stone and coworkers have shown that mice platelets are the predominant Adenovirus binding blood cell type and have documented the virus inside platelets [5] but the presence of CAR on mice platelets is yet to be verified. On the other hand, thrombocytopenia is well known in association with human viral infections including adenovirus and CAR has been identified on human platelets by Othman and coworkers [6] but this was contradicted by Shimony and coworkers in another study published in this journal [7]. The presence of CAR remains to be the most reasonable explanation of how Ad's entry into platelets takes place.

Platelets are known to express GP IIb/IIIa also known as αIIbβ3 which has a major role in platelet function particularly aggregation and interaction with other cells [8]. Platelets become activated rapidly following intravenous injection, Ad is seen inside platelets and the Ad-loaded platelets are taken up by Kupffer cells and are cleared from the circulation [5]. However, the kinetics of the platelet activation and which components of platelets involved in the internalization process remain unclear.

Since confirmation of the presence of CAR on the platelets is a key to the understanding of platelet adenovirus interaction, we were interested to re investigate this debate to confirm its presence on human platelets.

Human platelets were obtained from blood of healthy volunteers (2 weeks medication free prior to blood withdrawal) following their informed consent. Blood was collected from the anticubital vein using a 21-G needle into citrated tubes and gently mixed. PRP was prepared by centrifugation at 170 g for 17 min at RT and platelet count was adjusted to 2-3 × 10^6 ^platelets/μL. We tested platelets using rabbit H-300 polyclonal anti-CAR antibody and we included appropriate controls (CAR positive; HeLa cells and CAR negative; CHO cells) using fluorescence microscopy. 500 μl PRP was fixed in 1 ml 4% paraformaldehyde 10 min at RT and the platelet pellet was rinsed three times in PBS. Platelets were next incubated with 100 μl rabbit H-300 polyclonal anti-CAR antibody at 1:50 dilution for 1.5 hour, 37°C, washed three times in PBS, then incubated with 100 μl goat anti-rabbit IgG F(ab')_2 _Texas Red secondary antibody (Santa Cruz Biotechnology) in 1:100 dilution for 30 min, 37°C. Platelets were washed three times in PBS before applying to a drop of glycerol -gelatine on a glass slide and analyzed with fluorescent microscopy (Leica DM5000B). For quantitative analysis, ten random fields were selected and the number of platelets stained with Texas Red was counted relative to the total number of platelets and expressed as a percentage (the total number of platelets counted was no less than 1000 in any experiment).

In agreement with what was previously shown [6], human platelets were found to express CAR on their surface (Figure 1). Interestingly, the data in this study showed that not all of platelet population is CAR-positive. In contrast to the previous study by Othman et al., 2007, which showed > 70% of human platelet express CAR, only 3.5 ± 1.9% of the tested platelets did express CAR in the current study. In addition, CAR was seen within intracellular aggregates localized at the sites of cell-cell contacts indicating that CAR expression might be upregulated in response to platelet stimulation. The difference in the level of expression between the two studies can be explained on the basis of the platelet population tested. In the previous study, platelets from concentrates were used. These platelets are likely to be activated during handling and testing which can possibly lead to upregulating CAR hence the high percentage. In the current study, fresh human platelets form healthy volunteers were used.

**Figure 1:**
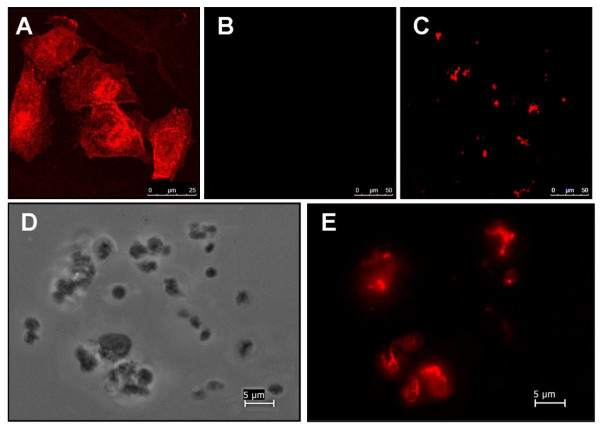
**Visualization of CAR on human platelets' surface**. CAR expression was tested on normal human platelets as well as positive and negative controls (CAR-positive (HeLa) and CAR-negative (CHO) cell lines). A, B, C, E) fluorescence microscopy, D - light microscopy. All cells were stained for CAR using rabbit H-300 polyclonal anti-CAR and goat anti-rabbit IgG F(ab')_2 _Texas Red- secondary antibodies as detailed above. A) CAR-positive Hela cells stained for CAR. B) CAR-negative (CHO) cells stained for CAR. C) human platelets stained for CAR. D, E) light view and fluorescent view (respectively) of the same sample of human platelets.

In the current study, we verified the presence of CAR on human platelets using the same anti CAR antibody used by Shimony et al., 2009; which reported an opposite finding (No CAR on platelets). The difference between our experiments and that of Shimony and coworkers can be explained by factors related to the PRP preparation. In their study, the authors excluded the platelet aggregates from the tested population and they adopted centrifugation at 5000 rpm for 4 minutes in the presence of citrate (5 mM) before platelet fixation. Such centrifugation may lead to platelet degranulation and structural alteration which makes it difficult to test or judge platelet surface proteins since the platelets are not in the native state.

A novel finding in the current study is that we found most of the CAR expression was localized to small platelet aggregates and at the sites of intracellular communication supporting the idea that CAR may be upregulated by platelet activation.

We conclude that human platelets express CAR and that this receptor is localized at the sites of intercellular interaction and is likely upregulated by platelet activation.

## Competing interests

The authors declare that they have no competing interests.

## Authors' contributions

EG designed research, performed experiments, analyzed research and wrote the paper. LB designed research, analyzed research and wrote the paper. MA designed research, analyzed research and wrote the paper. All authors read and approved the final manuscript.
